# 946. Epidemiology and Long-term Outcomes of BK Polyomavirus Nephropathy in Kidney Solid Organ Transplant Recipients at Texas Children’s Hospital

**DOI:** 10.1093/ofid/ofab466.1141

**Published:** 2021-12-04

**Authors:** Kristen Valencia Deray, Kathleen Hosek, Daniel Ruderfer, Sarah J Swartz, Claire Bocchini

**Affiliations:** 1 Baylor College of Medicine and Texas Children’s Hospital, Houston, TX; 2 Texas Children’s Hospital, Houston, TX; 3 Baylor College of Medicine/Texas Children’s Hospital, Houston, Texas; 4 Baylor College of Medicine, Houston, TX

## Abstract

**Background:**

BK Polyomavirus (BKPyV) is an important cause of graft dysfunction and premature graft failure in pediatric kidney transplant recipients (PKTR). Contemporary data on BK viral associated nephropathy (BKVAN) in PKTR are limited. We sought to determine the frequency, associations with, and long-term outcomes of BKVAN in PKTR.

**Methods:**

A retrospective cohort study of PKTR ≤21 years of age transplanted from 2011-2018 was completed. Primary outcome was BKVAN and secondary outcomes included graft dysfunction and failure. Associations with BKVAN were measured using chi square and Fisher exact tests. Time to BKVAN and graft failure were assessed using Kaplan-Meier plots.

**Results:**

Among 200 PKTR, 16 (8%) developed BKVAN at a median of 228 days post-transplant. Median (IQR) age at time of transplant for patients with BKVAN was 14 (7-17) years of age. Of those who developed BKVAN, 13/16 (81%) were biopsy proven, 2/16 (13%) were probable and 1/16 (6%) was presumptive. Treatment of BKVAN included reduced immunosuppression (12, 75%), ciprofloxacin (11, 69%), intravenous immunoglobulin (7, 44%), and leflunomide (4, 25%). Simultaneous rejection therapy occurred in two patients (13%). Notably, three patients with BKVAN had negative BKPyV plasma viral loads. Median (IQR) BKPyV viral load in those with positive PCRs was 82,000 (19,315 – 1,106,283) copies/milliliter. Median (IQR) time to clearance of BKPyV from the plasma was 425 (261 – 858) days. There was no association between age at time of transplant, repeat kidney transplant, donor type, underlying diagnosis at time of transplant, HLA mismatch, mode of dialysis, or steroid free immunosuppression and the development of BKVAN. Mean percent change in eGFR yearly post-transplant was -0.066 for those with BKVAN versus -0.091 for those without BKVAN (p=0.35). Graft failure was experienced in 1/16 (6%) PKTR with BKVAN but was not related to BKVAN. There was no difference in time to graft failure (Figure 1, p=0.64) in those who developed BKVAN versus those who did not.

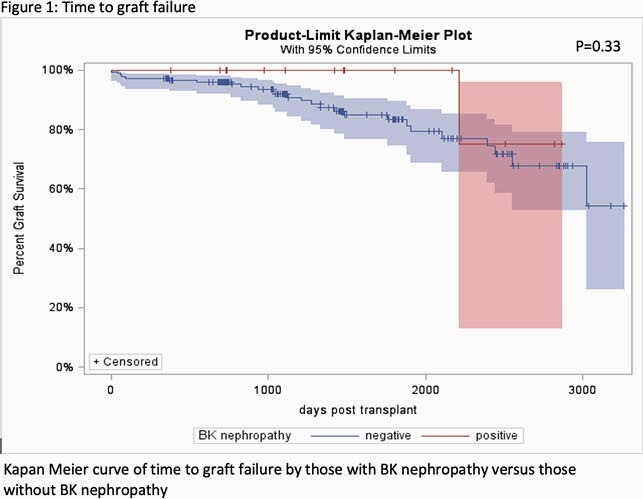

**Conclusion:**

BKVAN continues to occur in PKTR. No associations were found with the development of BKVAN in our cohort. PKTR with BKVAN did not have an increased rate of eGFR decline nor did they develop graft failure more quickly than those without BKVAN.

**Disclosures:**

**Sarah J. Swartz, MD**, **Education grant sponsored by Amgen; awarded by Renal Physicians Association** (Other Financial or Material Support, Attended Renal Physician Association PAL (Pediatric Advocacy Leadership) Forum in Jun 2019; Program sponsored by Education grant from Amgen, awarded by the Renal Physicians Association for participation)**Vifor Fresensius Medical Care Renal Pharma** (Scientific Research Study Investigator, Multi-center site PI for phase 3 study to investigate the safety and efficacy of PA21 (Velphoro) and calcium acetate (Phoslyra) sponsored by Vifor Fresensius Medical Care Renal Pharma funded Sept 2016-Oct 2019)

